# Malaria Risk Factors in North West Tanzania: The Effect of Spraying, Nets and Wealth

**DOI:** 10.1371/journal.pone.0065787

**Published:** 2013-06-07

**Authors:** Philippa A. West, Natacha Protopopoff, Mark Rowland, Emma Cumming, Alison Rand, Chris Drakeley, Alexandra Wright, Zuhura Kivaju, Matthew J. Kirby, Franklin W. Mosha, William Kisinza, Immo Kleinschmidt

**Affiliations:** 1 Department of Infectious Disease Epidemiology, London School of Tropical Medicine and Hygiene, London, United Kingdom; 2 Department of Disease Control, London School of Tropical Medicine and Hygiene, London, United Kingdom; 3 Immunology and Infection Department, London School of Tropical Medicine and Hygiene, London, United Kingdom; 4 National Institute for Medical Research, Amani Medical Research Centre, Muheza, Tanzania; 5 Kilimanjaro Christian Medical College, Tumaini University, Moshi, Tanzania; 6 MRC Tropical Epidemiology Group, London School of Tropical Medicine and Hygiene, London, United Kingdom; Tulane University School of Public Health and Tropical Medicine, United States of America

## Abstract

Malaria prevalence remains high in many African countries despite massive scaling-up of insecticide treated nets (ITN) and indoor residual spraying (IRS). This paper evaluates the protective effect of pyrethroid IRS and ITNs in relation to risk factors for malaria based on a study conducted in North-West Tanzania, where IRS has been conducted since 2007 and universal coverage of ITNs has been carried out recently. In 2011 community-based cross-sectional surveys were conducted in the two main malaria transmission periods that occur after the short and long rainy seasons. These included 5,152 and 4,325 children aged 0.5–14 years, respectively. Data on IRS and ITN coverage, household demographics and socio-economic status were collected using an adapted version of the Malaria Indicator Survey. Children were screened for malaria by rapid diagnostic test. In the second survey, haemoglobin density was measured and filter paper blood spots were collected to determine age-specific sero-prevalence in each community surveyed. *Plasmodium falciparum* infection prevalence in children 0.5–14 years old was 9.3% (95%CI:5.9–14.5) and 22.8% (95%CI:17.3–29.4) in the two surveys. Risk factors for infection after the short rains included households not being sprayed (OR = 0.39; 95%CI:0.20–0.75); low community net ownership (OR = 0.45; 95%CI:0.21–0.95); and low community SES (least poor vs. poorest tertile: OR = 0.13, 95%CI:0.05–0.34). Risk factors after the long rains included household poverty (per quintile increase: OR = 0.89; 95%CI:0.82–0.97) and community poverty (least poor vs. poorest tertile: OR = 0.26, 95%CI:0.15–0.44); household IRS or high community ITN ownership were not protective. Despite high IRS coverage and equitable LLIN distribution, poverty was an important risk factor for malaria suggesting it could be beneficial to target additional malaria control activities to poor households and communities. High malaria prevalence in some clusters and the limited protection given by pyrethroid IRS and LLINs suggest that it may be necessary to enhance established vector control activities and consider additional interventions.

## Introduction

An estimated 17% global reduction in malaria incidence has been achieved between 2000 and 2010, however despite this progress 174 million episodes of malaria were estimated to have occurred in Africa alone in 2010 [1]. Insecticide treated nets (ITN) and indoor residual spraying (IRS) have both been demonstrated to reduce malaria [2], [3], [4], [5], [6], [7]. In line with the Roll Back Malaria Abuja declaration in 2000 [8] ITN and IRS have been scaled-up in Africa over the past decade. The WHO World Malaria Report 2012 reported that in Africa in 2011 53% of households owned an ITN and IRS protected 11% of the population at risk [9]. Mass distribution campaigns delivering LLINs free of charge is one of the strategies recommended by WHO in malaria risk areas [10].

Thirty-one (72%) endemic countries in Africa were reported to use both IRS and ITN in at least some areas in 2010 [1]. However, it is unclear from current evidence whether the combined use of LLINs and IRS provide an additional benefit to using either intervention alone, and if this benefit will be seen in all epidemiological situations [11], [12]. There are two ways that the combined interventions can improve protection against malaria. Firstly, people who are not protected by one intervention may be protected by the other; this is particularly important where coverage is low. Secondly, an individual using both interventions may receive additional protection above that from using one alone [12]. There have been two theoretical models that suggest that the combination could reduce malaria transmission faster and further than one intervention alone [13], [14]. A third model by Yakob and colleagues also reported a benefit from the combination in some settings but suggested that the combination could work antagonistically in certain cases [15]. It is possible that IRS may reduce mosquito abundance in the houses and thus reduce the incentive to use ITNs.

In Tanzania the Malaria Indicator Survey of 2007–2008 found a malaria prevalence in children under five was 18.1% [16]. There has been a greater than 50% reduction in malaria admissions to health centres and hospitals between 2000 and 2010, which coincides with the scaling-up of malaria control activities. ITN have been distributed since 2005 and a universal coverage campaign of LLIN was completed in 2011. IRS funded by the President’s Malaria Initiative (PMI) started in 2007 in two districts of Kagera region, Northwest Tanzania. Since 2009 the IRS programme has been extended to include 18 districts around lake Victoria [17].

Establishing determinants of infection and evaluating the effectiveness of vector control interventions can identify possible ways to improve malaria control. This study uses observational data from two large cross sectional surveys conducted in 2011 to investigate the protective effect of LLINs and IRS in relation to risk factors for malaria infection in Muleba district, North-West Tanzania.

## Methods

### Study Area

The study area has been described elsewhere [18]. In summary, the study was carried out in 68 rural villages in Muleba district (1? 45′ S 31? 40′ E), in Kagera region on the western shore of Lake Victoria, North-West Tanzania. There are on average 4.3 hamlets per village, 146 households per hamlet and 5.5 individuals per household. The study area is situated at 1100–1600 m above sea level, includes 68,108 households and covers approximately two thirds of Muleba district. A very high proportion (93.8%) of the Kagera population live in rural areas and 66.9% of the population over 10 years old were estimated to be literate; slightly lower than the national average of 69.8% [19].

The National Malaria Indicator Survey 2007–2008 reported a malaria prevalence of 41% in children under five in Kagera region [16]. The Malaria Atlas Project estimated Muleba district to have a 5–40% *Plasmodium falciparum* parasite rate [20]. Kagera region faced a major malaria epidemic between 1997 and 1998 [21]. It was reported that Muleba experienced outbreaks in 2006 [22] and 2010 (Research Triangle Institute unpublished data). There are two annual rainy seasons; the short rains in October-December (average monthly rainfall 160 mm) and the long rains in March-May (average monthly rainfall 300 mm) [23]. Malaria transmission occurs throughout the year and peaks after the two rainy seasons [24].

The trial area was divided into 50 clusters following the mapping and enumeration of every household with hand held Global Positioning System devices (Garmin *e*trex legend H®, Garmin International Inc. USA). Each cluster was composed of a buffer zone at least 1 km wide surrounding a core sampling area approximately 1 km in radius where the surveys were conducted; the core areas were comprised of between one and five hamlets with a minimum of 200 households.

### The Interventions

Annual rounds of indoor residual spraying (IRS) have been conducted in Muleba district with the pyrethroid lambdacyhalothrin (ICON®10CS, Syngenta, Basel, Switzerland) between 2007 and 2011. Spraying was carried out by Research Triangle Institute (RTI) with funding from PMI. At the time of the surveys for this study, the villages had been sprayed four or five times depending on year of enrolment, most recently in January-February 2011. The other intervention is high coverage of LLINs. Tanzania has a national pregnant women voucher scheme (TNVS) for the routine distribution of LLINs. LLIN coverage has been increased further by two mass distribution campaigns; the first targeting children under five in 2009/2010 [25] and the second in 2011 aiming to achieve universal coverage of ITNs (one ITN per sleeping place). After the universal coverage campaign (UCC) 91% of households owned at least one ITN [18].

### Surveys

Two household cross-sectional surveys were undertaken to collect data in the two annual malaria transmission seasons. Survey one (22^nd^ February-25^th^ March 2011) was conducted after the short rainy season and survey two (27^th^ June –3^rd^ August 2011) after the long rainy season. The first survey was approximately one month after IRS and immediately before the UCC and the second was approximately five months after IRS and three months after the UCC. These surveys form the baseline assessment for an ongoing cluster randomised control trial (CRT) to compare the effect of the combined use of IRS and LLINs versus LLINs alone on malaria transmission (clinical trial registration number NCT01697852).

One hundred households per cluster were randomly selected in the first survey and 80 in the second. Households with children aged 6 months to 14 years were eligible for inclusion. Household heads or other resident adults were interviewed in either Kiswahili (national language) or Kihaya (local language) after written informed consent had been obtained. Data on IRS coverage, bed net ownership and usage, demographics of household members, and other household characteristics including factors related to socio economic status (SES) were gathered using an adapted version of the standard Malaria Indicator Survey [26]. Questions regarding SES and acceptability of IRS and ITNs were only collected in survey two.

A maximum of two children per household were selected at random and clinically examined the following day at a central location in the village. Axillary temperature and thick and thin blood smears were taken, and rapid diagnostic tests (RDT) for malaria parasites were conducted (Survey 1: Paracheck-*P. falciparum*, Orchid Biomedical Systems, Goa, India and Survey 2: CareStart™ Malaria DiaSys, Wokingham, UK). In survey two, haemoglobin densities were measured (HemoCue® Hb 201^+^, Aktiebolaget Leo Diagnostics, Helsingborg, Sweden) and filter paper blood spots were collected for the detection of antibodies to *P. falciparum* blood stage antigen. Cases with malaria infection detected by RDTs were treated with artemisinin-based combination therapy (ACTs). For serological analysis, in survey two, all pregnant women, plus one adult randomly selected from every third selected household were clinically examined.

### Laboratory Methods

A standard ELISA was used to detect antibodies to *P. falciparum* asexual stage merozoite antigen, glutamate rich protein R2 (GLURP) and membrane surface protein (MSP-1_19_) from blood spots from all study children and a sample of individuals aged over 14 years old [27]. Optical density values were averaged and normalised to a standard value of a hyperimmune control serum [28]. Cut off values to define sero-prevalence (0.216 for GLURP and 0.188 for MSP-1_19_) were calculated using the mixture model function [28] in STATA 12 (STATAcorp, Texas, USA).

### Statistical Analysis

Data were collected by interviewers using Pendragon™ Forms (Pendragon Corporation Software, Libertyville, USA) on Personal Digital Assistants (PDA), and then transferred into a Microsoft Access database (Microsoft Corporation, Redmond, USA). All statistical analysis was done in STATA 12 (STATAcorp, Texas, USA).

For the purpose of this analysis ‘ITNs’ was used to refer to LLINs, pre-treated nets that were less than 1 year old and nets that had been re-treated within the last year.

*Plasmodium falciparum* malaria infection was defined as *P. falciparum* alone or as a mixed species infections as detected by RDT. *Plasmodium falciparum* infection prevalence was calculated for the study area overall and by cluster. Unless otherwise stated analysis was completed and the results are given for children aged 6 months to 14 years. SES quintiles were created using Principle Component Analysis of the following household characteristics: number of rooms, household crowding, level of schooling of the household head, type of house (including floor, wall, and roof materials) and ownership of livestock, farmland, bicycles, mobile phones and radios [29], [30]. Community SES was estimated from the mean household SES of the cluster: clusters were grouped into tertiles of SES score.

For all children and separately those aged less than five years study site and cluster level anaemia prevalence estimates (adjusting for altitude) were calculated for: moderate to severe (<8 g/dL) and any anaemia (cut off varied with age) [31], [32]. Logistic regression that allowed for clustering was used to calculate the odds of moderate to severe anaemia by *P. falciparum* infection status.

Evaluations of current control programmes generally use observational data as trials cannot be feasibly conducted. One problem with this approach to evaluation is that the uptake of vector control measures is sometimes highest in the areas with the highest risk of malaria. Serology has been used historically [4], [33] and more recently [27], [34], [35], [36] to assess transmission intensity. Sero-conversion rates were used to adjust for transmission intensity when evaluating the effectiveness of control measures using cross-sectional data in a study on Bioko Island, Equatorial Guinea [37]. In the present study cluster sero-prevalence was used to adjust for recent malaria exposure. In this analysis sero-positive was considered as positive to either antigen (GLURP or MSP-1_19_). Age standardised sero-prevalence was calculated for each cluster using direct standardisation against the total population studied.

Risk factors for individual *P. falciparum* infection in children 6 months to 14 years old were analysed for each survey using logistic regression allowing for clustering using the survey commands in STATA 12. Sero-prevalence data was only available for 44 out of the 50 clusters thus only these were included in the risk factor analysis. Household altitude, housing density at the cluster level and mean cluster altitude were not included in the final models as they were not confounders for the risk factors of interest and they are on the causal pathway for cluster sero-prevalence.

### Risk Factors Investigated

•Individual level factors: Age, gender, slept under an ITN the previous night

•Household level factors:

- Crowding, number residents, SES quintiles (survey 2 only),
- Household ITN ownership (at least one ITN per two residents)
- Household ITN usage (number of ITNs used, number of individuals under ITNs, all residents under an ITN)
- IRS in 2011

•Cluster level factors:

- Age-adjusted sero-prevalence (from the survey 2 as a proxy for transmission intensity)
- Community SES (from survey two)
- IRS coverage (from survey one approximately one month after spraying)
- Proportion of cluster residents that used an ITN the previous night
- Proportion of households with adequate ITNs (at least one ITN per two residents)

All factors associated with *P. falciparum* infection with a p-value of <0.2 were fitted to a multi variable logistic regression model using the step-wise technique and retained if the adjusted p-value was <0.05. It was pre-specified that interactions between malaria transmission intensity and the following factors would be investigated: household IRS; ITN use the previous night; and any factors relating to IRS or ITN use that were independently associated with *P. falciparum* infection.

### Ethics Statement

The trial was approved by the ethics review committees of the Kilimanjaro Christian Medical College, the National Institute for Medical Research Tanzania and the London School of Hygiene and Tropical Medicine. Written informed consent was obtained from all participants or the parents/guardians if under 15 years old.

## Results

### Selection and Characteristics of Study Participants

**Figure 1:**
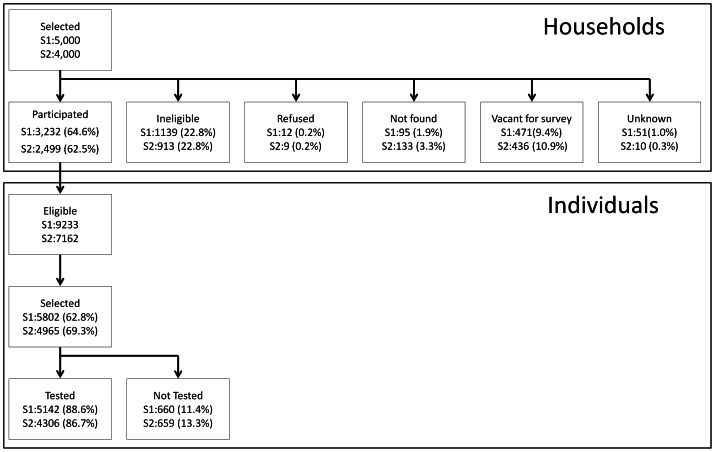
Study profile: Household and participant selection and participation in survey one (S1) and two (S2)

In survey one 5,000 households were selected, of which 64.6% were eligible with children aged 0.5–14 years and consented to participate in the study. Twelve households (0.2%) refused to participate. There were 9,233 children aged between 6 months and 14 years, of these 5,802 were selected for inclusion and of whom 88.6% participated (Figure 1). The percentages included in the study at each step in survey two were consistent with study one except that an additional 899 individuals aged older than 14 years were tested; 177 of these were pregnant women. **Table 1** illustrates that the demographics of the participants in the two surveys are broadly similar. Net ownership and usage was higher in survey two after the UCC (**Table 1** and **Table 2**).

**Table 1:** Individual and household characteristics of the study participants in Muleba region, Tanzania, 2011.

	SURVEY 1^1^		SURVEY 2^2^	
	Distribution of characteristics	*Pf* infection prevalence^3^	Distribution of characteristics	*Pf* infection prevalence^3^
	% (N)	[95% CI],(N)	% (N)	[95% CI],(N)
**Study Children (0.5–14 years)**	**100 (5152)**		**100 (4325)**	
**Gender**				
Male	49.6 (2535)	9.5 [5.9–15.0],(2526)	49.4 (2134)	22.6 [16.9–29.6],(2124)
Female	50.4 (2575)	9.1 [5.7–14.4],(2574)	50.6 (2183)	23.0 [17.6–29.5],(2174)
**Age (years)**				
0.5–<1 years	4 (204)	5.4 [2.1–13.1],(204)	3.8 (165)	15.2 [10.9–20.9],(164)
1–4 years	35 (1805)	10.4 [6.5–16.1],(1801)	34.1 (1473)	23.7 [17.5–31.3],(1468)
5–9 years	36.1 (1858)	10.7 [6.7–16.8],(1854)	36.1 (1562)	24.5 [18.4–31.7],(1554)
10–14 years	24.9 (1285)	6.5 [4.0–10.3],(1283)	26.0 (1125)	20.3 [15.1–26.6],(1120)
**ITN used the previous night**				
No	56.6 (2915)	8.1 [5.0–13.0],(2911)	41.9 (1811)	20.3 [15.1–26.8],(1805)
Yes	43.4 (2237)	10.9 [6.8–17.0],(2231)	58.1 (2514)	24.5 [18.5–31.7],(2501)
**Study Households**	**100 (2936)**		**100 (2215)**	
**Household size** 4				
Mean [CI]	5.5 [5.4–5.6]		5.6 [5.5–5.7]	
2–4 residents	34.7 (1018)	9.0 [5.8–13.8],(1514)	30.2 (670)	22.8 [17.3–29.4],(987)
5–6 residents	37.6 (1105)	8.8 [5.3–14.3],(2053)	40.5 (896)	23.4 [17.4–30.8],(1867)
7–15 residents	27.7 (813)	10.3 [6.0–17.2],(1575)	29.3 (649)	21.9 [16.4–28.7],(1452)
**Household crowding** 5				
<1 resident per room	NO DATA*		39.7 (878)	22.8 [17.3–29.4],(987)
≥1 resident per room			60.3 (1331)	22.7 [17.1–29.6],(3319)
**Household SES** 6				
*Poorest*			19.6 (424)	33.1 [25.4–41.8],(768)
*Very poor*			20.0 (434)	28.1 [21.0–36.5],(826)
*Poor*	NO DATA*		20.3 (440)	23.4 [17.0–31.1],(865)
*Less poor*			20.4 (441)	17.3 [12.7–23.0],(886)
*Least poor*			19.7 (426)	13.3 [9.1–18.9],(859)
**Distance to health facility (km)**				
Mean [CI]	3.7 [3.0–4.3]		3.7 [3.0–4.3]	
0–2.4 km	30.3 (882)	6.7 [2.4–17.2],(1529)	29.3 (649)	15.0 [8.2–25.8],(1188)
2.5–3.9 km	35.7 (1040)	7.5 [4.3–12.8],(1841)	36.4 (807)	20.7 [13.8–29.9],(1572)
≥4 km	34 (991)	13.5 [7.2–24.0],(1746)	34.3 (759)	30.8 [22.7–40.2],(1546)
**Household sprayed** 7				
No	4.7 (137)	20.9 [10.0–38.6],(230)	6.0 (132)	25.2 [15.4–38.3],(250)
Yes	95.3 (2792)	8.8 [5.5–13.8],(4899)	94.0 (2076)	22.5 [17.1–29.1],(4042)
**Household owns ≥1 ITN**				
No	36.6 (1074)	7.9 [4.7–13.1],(1736)	8.2 (182)	25.6 [17.5–35.8],(336)
Yes	63.4 (1862)	10.0 [6.3–15.6],(3406)	91.8 (2033)	22.5 [17.0–29.3],(3970)
**Household ITN ownership** 8				
No	87.4 (2565)	9.7 [6.1–15.1],(4500)	64.9 (1437)	22.9 [17.3–29.8],(2878)
Yes	12.6 (371)	6.7 [3.5–12.4],(642)	35.1 (778)	22.4 [16.4–29.9],(1428)

Note: CI = confidence intervals, N = number in each category,

1 22nd February- 25th March 2011,

2 27th June- 3rd August 2011,

3 *Plasmodium falciparum* infection prevalence in children 0.5–14 years old from RDTs,

4 number of residents,

5 residents per room,

6 SES quintiles

7 Sprayed in January 2011-Feb 2011,

8 households owning ≥1 ITN per 2 residents (universal coverage),

* questions not included in survey one.

**Table 2:** Characteristics of the study clusters in Muleba region, Tanzania, 2011.

	SURVEY 1^1^		SURVEY 2^2^	
	Distribution of characteristics	*Pf* infection prevalence^3^	Distribution of characteristics	*Pf* infection prevalence^3^
	% (N)	% [95% CI], (N)	% (N)	% [95% CI], (N)
**Study Clusters**	100 (50)		100 (50)	
**Housing density** 4				
*<100 HH/km* 2	24.0 (12)	27.9 [19.3,38.6],(1218)	24.0 (12)	49.9 [44.8,54.9],(1151)
*100*–*163 HH/km* 2	26.0 (13)	7.4 [2.7,18.7],(1325)	26.0 (13)	22.5 [13.8,34.3],(1109)
*164*–*199 HH/km* 2	28.0 (14)	2.2 [0.9,5.2],(1428)	28.0 (14)	9.0 [6.7,11.9],(1171)
≥*200 HH/km* 2	22.0 (11)	0.9 [0.4,1.6],(1171)	22.0 (11)	5.9 [3.8,9.2],(875)
**Altitude** 5				
1150–1249 m	30.0 (15)	19.1 [10.6,32.0],(1511)	30.0 (15)	37.2 [28.3,47.0],(1363)
1250–1349 m	30.0 (15)	9.8 [4.9,18.4],(1564)	30.0 (15)	26.3 [16.8,38.6],(1270)
1350–1449 m	10.0 (5)	5.2 [2.5,10.6],(537)	10.0 (5)	17.6 [8.3,33.8],(425)
≥1450 m	30.0 (15)	0.7 [0.3,1.5],(1530)	30.0 (15)	5.1 [3.8,6.8],(1248)
**Spray coverage** 6				
<90%	16.0 (8)	18.9 [8.5,36.9],(736)	16.0 (8)	30.5 [17.0,48.6],(655)
90%–<95%	16.0 (8)	2.3 [0.7,7.3],(833)	16.0 (8)	15.2 [6.5,31.7],(671)
≥95%	68.0 (34)	9.0 [5.1,15.4],(3573)	68.0 (34)	22.8 [16.4,30.7],(2980)
**ITN usage** 7				
<40%	46.0 (23)	4.3 [1.7,10.3],(2301)	8.0 (4)	8.5 [4.8,14.4],(342)
40–54%	48.0 (24)	12.9 [7.6,21.0],(2579)	28.0 (14)	18.0 [9.9,30.6],(1137)
≥55%	6.0 (3)	18.7 [2.8,64.6],(262)	64.0 (32)	26.4 [19.3,34.9],(2827)
**ITN ownership** 8 **: Survey 1**				
≤15% of households	72.0 (36)	10.8 [6.5,17.4], (3961)	NOT APPLICAPLE	
>15% of households	28.0 (14)	4.5 [1.8,10.9], (1181)		
**ITN ownership** 8 **: Survey 2**				
<30% of households	NOT APPLICAPLE		34.0 (17)	23.7 [15.4,34.8],(1857)
≥30% of households			64.0 (32)	22.0 [15.3,30.6],(2449)
**Sero-prevalence** 9	100 (44)		(44)	
0–19%	34.1 (15)	0.8 [0.4,1.7],(1558)	34.1 (15)	4.8 [3.7,6.2],(1296)
20–39%	31.8 (14)	3.0 [2.0,4.5],(1399)	31.8 (14)	19.0 [13.0,26.8],(1128)
≥40%	34.1 (15)	26.3 [17.8,36.8],(1539)	34.1 (15)	46.9 [40.0,53.9],(1403)

Note: N = number in each category, CI = confidence intervals,

1 22nd February- 25th March 2011,

2 27th June- 3rd August 2011,

3 *Plasmodium falciparum* infection prevalence in children 0.5–14 years old from RDTs,

4 Households per square Km,

5 mean altitude of all households mapped in the core surveillance area of the cluster (metres above sea level),

6 Sprayed in January 2011-Feb 2011 and recorded in survey 1,

7 Percentage of all residents that slept under an ITN the previous night,

8 Percentage of households owning ≥1 ITN per 2 residents (universal coverage),

9 age adjusted. Altitude and housing density were not included in the multivariable model as they were not confounders for the main variables of interest.

### Prevalence of P. falciparum Infection, Clinical Malaria, Anaemia and Sero-prevalence

**Figure 2:**
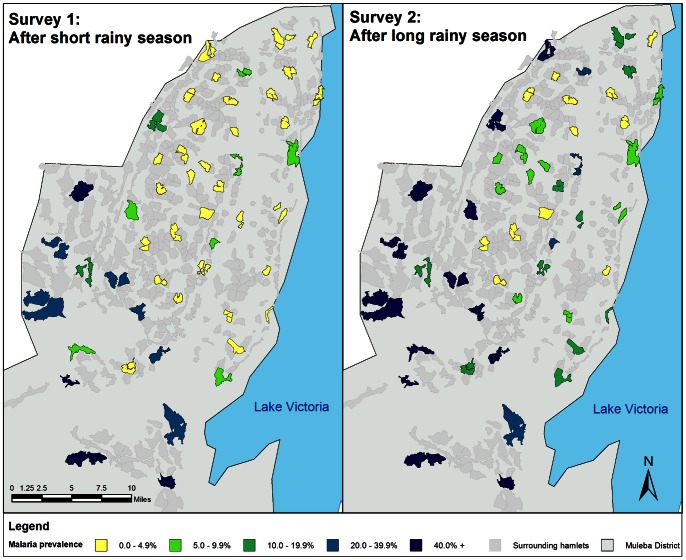
*Plasmodium falciparum* infection prevalence by cluster for survey one and two. Note: *Plasmodium falciparum* infection from RDT in children 0.5-14 years old in Muleba district, Tanzania, 2011.

*Plasmodium falciparum* infection prevalence determined by RDTs in children aged 6 months to 14 years old was 9.3% (95% CI 5.9–14.5) in survey one and 22.8% (95% CI 17.3–29.4) in survey two. **Figure 2** shows that *P. falciparum* infection prevalence was heterogeneous between clusters. Cluster estimates ranged from 0 to 55.7% in survey one and 1.3 to 61.5% in survey two. The maps (**Figure 2**) show that the highest transmission was in the south-west of the district. *Plasmodium falciparum* infection prevalence increased with age, reaching a peak in six year olds at 11.6% in survey one and 28.9% in survey two. In survey two 9.6% (95% CI 5.9–15.2, n = 177) of pregnant women and 10.3% (95% CI 7.4–14.2, n = 719) of non-pregnant adults (aged over 14 years) had *P. falciparum* infection.

The prevalence of clinical malaria – defined as fever (axillary temperature ≥37.5?C) and *P. falciparum* infection – in children was 4.7% (95% CI = 2.7–8.2, N = 5147) and 4.3% (95% CI = 2.9–6.4, N = 4319) in the first and second survey respectively. Of children with *P. falciparum* infection, 50.7% (95% CI = 33.4–67.9%) had fever in survey one and 18.7% (95% CI = 12.6–26.9%) in survey two. The odds of fever were 62.1 (95% CI 25.9–148.9) times higher in those that were *P. falciparum* positive compared to *P. falciparum* negatives in survey one and 18.9 (95% CI 9.7–36.7) times higher in survey two. Amongst children with *P. falciparum,* the percentage of fever cases that could be attributed to *P. falciparum* infection (attributable fraction) were 96.8% and 93.5% in survey one and two respectively. Of all fever cases in the community 73.7% and 76.7% could be attributed to *P. falciparum* infection in survey one and two [38]. The association between fever and *P. falciparum* infection does not vary with age (interaction p-value survey 1 = 0.28 and survey 2 = 0.45). Of the children that had fever, 76.2% (95% CI 58.3–88.0%) and 75.2% (95% CI = 63.5–84.1%) were *P. falciparum* positive in survey one and two respectively.

Anaemia prevalence data was only collected in survey two. In children under 5 years 55% (95% CI = 50.8–59.2%, N = 1626) had some level of anaemia and 6.2% (95% CI = 4.5–8.5) had moderate to severe anaemia (<8 g/dL). Moderate-severe cluster estimates ranged from 0 to 23.8%. The odds of moderate-severe anaemia were eight times higher if the child had *P. falciparum* infection (18.4% vs. 2.6%, OR = 8.3, 95% CI = 5.3–13.2).

**Figure 3:**
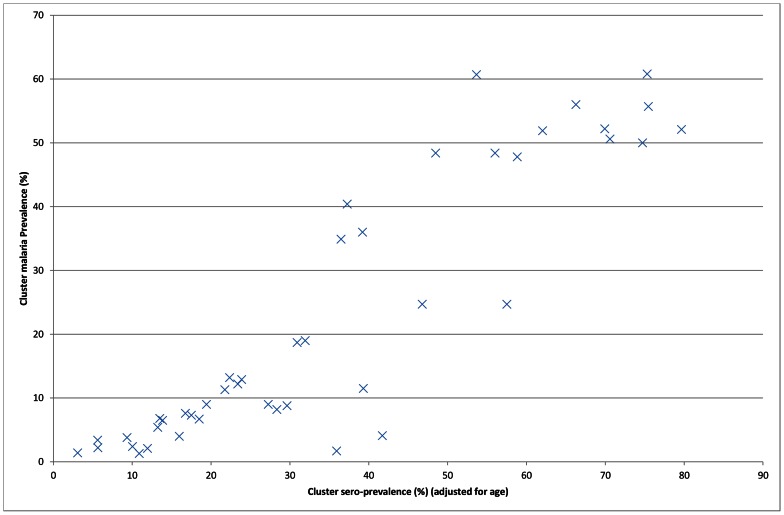
Cluster *Plasmodium falciparum* infection by cluster sero-prevalence in survey two in Muleba district, Tanzania, 2011. Note: *Plasmodium falciparum* infection from RDT in children 0.5-14 years old in survey two. Sero-prevalence is from survey two and is adjusted for age. There is one point per cluster.

Total sero-prevalence (sero-positive for either MSP-1_19_ or GLURP) in all residents was 37.3% (95% CI = 35.9–38.7%). Cluster estimates (adjusted for age) ranged from 3.1% to 79.7% (**Figure 3**). There was a positive association between cluster sero-prevalence and cluster *P. falciparum* infection (**Figure 3**). In children the odds of *P. falciparum* infection were 12.2 times higher for sero-positives. Sero-prevalence was 72.8% and 18% in children with and without *P. falciparum* infection (RDT) respectively.

### Risk Factors for *P. falciparum* Infection

In survey one, *P. falciparum* infection of individuals was independently associated with age, household IRS status, community ITN ownership, community SES (defined from survey two), and malaria transmission intensity (cluster sero-prevalence defined from survey two) (Table 3). Compared to the baseline age group of children 10–14 years, the odds of *P. falciparum* infection were 1.6 times higher in the in the 5–9 year olds (OR = 1.6, 95% CI = 1.1–2.3). Infection was considerably lower in sprayed than unsprayed houses (OR = 0.39, 95% CI = 0.20–0.75). There was weak evidence (interaction p-value = 0.15) that the size of this effect was stronger in areas with low malaria transmission intensity (high transmission areas: OR = 0.13, 95% CI = 0.03–0.61 and low transmission areas: OR = 0.45, 95% CI = 0.22–0.90). The odds of *P. falciparum* infection were lower in clusters with more than 15% of households with one or more ITN per two residents (OR = 0.50, 95% CI = 0.32–0.78). Children in clusters in the highest SES tertile have a much lower odds of *P. falciparum* infection than those that live in the lowest tertile villages (OR = 0.13, 95% CI = 0.05–0.34). No other factors that were investigated were independently associated with *P. falciparum* infection including sleeping under an ITN the previous night. Household SES was not assessed in survey one.

**Table 3:** Factors independently associated with *P. falciparum* infection in children 6 m to 14 years old in Survey 1, Muleba district, Tanzania, February-March 2011.

	Pf infection	Unadjusted (50 clusters)		Adjusted Model^1^	
	% [95% CI], N	OR [95% CI]	p-value	OR [95% CI]	p-value
**Household sprayed** 2					
No	20.9 [10.0,38.6], (230)	1.00		1.00	
Yes	8.8 [5.5,13.8], (4899)	0.37 [0.17–0.81]	0.0145	0.38 [0.20–0.75]	0.0056
**Cluster ITN ownership** 3					
≤15% of households	10.8 [6.5,17.4], (3961)	1.00		1.00	
>15% of households	4.5 [1.8,10.9], (1181)	0.39 [0.13–1.18]	0.0924	0.50 [0.32–0.78]	0.0028
**Cluster SES** 4					
Poorest	23.1 [15.2,33.6], (1677)	1.00		1.00	
Mid	4.0 [1.7,9.4], (1772)	0.14 [0.05–0.40]	<0.0001	0.69 [0.34–1.40]	0.0006
Least Poor	1.2 [0.6,2.4], (1693)	0.04 [0.02–0.10]		0.13 [0.05–0.34]	
**Cluster Sero-prevalence** 5					
0–19%	0.8 [0.4,1.7], (1558)	1.00		1.00	
20–39%	3.0 [2.0,4.5], (1399)	3.68 [1.61–8.43]	<0.0001	3.21 [1.37–7.53]	<0.0001
≥40%	26.3 [17.8,36.8], (1539)	42.30 [17.84–100.30]		21.77 [8.12–58.34]	
**Age (years)**					
0.5–0.9 yrs	5.4 [2.1,13.1], (204)	0.82 [0.34–1.99]	<0.0001	0.53 [0.22–1.28]	0.0001
1–4 yrs	10.4 [6.5,16.1], (1801)	1.68 [1.23–2.29]		1.12 [0.80–1.58]	
5–9 yrs	10.7 [6.7,16.8], (1854)	1.74 [1.29–2.34]		1.64 [1.15–2.33]	
10–14 yrs	6.5 [4.0,10.3], (1283)	1.00		1.00	

Note: *Pf* = *Plasmodium falciparium* infection from RDT, OR = odds ratio, CI = confidence interval.

1 Adjusted for all other variables in the table and using data from 44 clusters with N = 4483.

2 Reported sprayed in January-February 2011,

3 Percentage of households with universal coverage of ITNs (≥1 ITN per 2 residents in the household),

4 Cluster SES = mean SES score obtained from survey two from all households in the cluster and grouped into tertiles,

5 Cluster sero-prevalence is adjusted for age and is from survey two.

In survey two, *P. falciparum* infection was independently associated with age, household SES, community SES and high malaria transmission intensity. The odds of infection were similar in children aged 1 to 14 years of age (Table 4). The odds of *P. falciparum* infection was inversely related to household SES (OR = 0.91 per SES quintile, 95% CI 0.83–0.99). The odds of *P. falciparum* infection in the least poor villages was approximately halved compared to the poorest villages (OR = 0.45, 95% CI = 0.25–0.82). The odds of P. falciparum infection were 1.5 times higher for each 10% rise in cluster sero-prevalence. No other factors that were investigated were independently associated with *P. falciparum* infection in survey two including: sleeping under an ITN the previous night, cluster ITN coverage nor whether the house was sprayed.

**Table 4:** Factors independently associated with *P. falciparum* Infection in children 6 m to 14 years old in Survey 2, Muleba district, Tanzania, June-July 2011.

	*Pf* infection	Unadjusted (50 clusters)		Adjusted Model^1^	
	% [95% CI], N	OR [95% CI]	p-value	OR [95% CI]	p-value
**Household SES** 2					
*Poorest*	33.1 [25.4,41.8], (768)				
*Very poor*	28.1 [21.0,36.5], (826)				
*Poor*	23.4 [17.0,31.1], (865)	0.75 [0.68–0.82]^3^	<0.0001	0.91 [0.83–0.99]	0.0225
*Less poor*	17.3 [12.7,23.0], (886)				
*Least poor*	13.3 [9.1,18.9], (859)				
**Cluster SES** 4					
*Poorest*	42.5 [34.4,51.0], (1475)	1.00		1.00	
*Poor*	16.9 [10.1,27.0], (1420)	0.28 [0.14–0.55]	<0.0001	0.90 [0.51–1.60]	0.0095
*Least Poor*	8.0 [5.4,11.6], (1411)	0.12 [0.07–0.20]		0.45 [0.25–0.82]	
**Cluster sero-prevalence** 5					
*0*–*19%*	4.8 [3.7,6.2], (1296)				
*20*–*39%*	19 [13.0,26.8], (1128)	1.63 [1.54–1.74]^6^	<0.0001	1.49 [1.35–1.65]	<0.0001
*> = 40%*	46.9 [40.0,53.9], (1403)				
**Age (years)**					
*0.5–0.9* *yrs*	15.2 [10.9,20.8], (164)	0.71 [0.50–1.00]	0.0024	0.37 [0.26–0.53]	<0.0001
*1–4* *yrs*	23.7 [17.5,31.3], (1468)	1.22 [0.99–1.51]		0.97 [0.78–1.22]	
*5–9* *yrs*	24.5 [18.4,31.7], (1554)	1.27 [1.06–1.53]		1.14 [0.91–1.43]	
*10–14* *yrs*	20.3 [15.1,26.6], (1120)	1.00		1.00	

Note: *Pf* = *Plasmodium falciparium* infection from RDT, OR = odds ratio, CI = confidence Interval.

1 Adjusted for all other variables in the table and using data from 44 clusters with N = 3736.

2 SES Quintiles obtained from survey two,

3 linear per quintile increase,

4 Cluster SES = mean SES score obtained from survey two from all households in the cluster and grouped into tertiles,

5 Cluster sero-prevalence is adjusted for age and is from survey two,

6 Linear per 10% increase.

### Vector Control Coverage and Acceptance

Approximately one month after the spraying (first survey) reported coverage was 95.3% (**Table 1**) and cluster level estimates ranged from 72.5% to 100%. Coverage was at least 90% in 42 out of 50 clusters. In survey two 95% of households reported wanting their houses to be sprayed in the next round. Protection against malaria was listed by 47% of households as the reason for agreeing to spraying, while 45% listed protection from mosquitoes. Householders reported that houses were usually not sprayed due to either the spray team not coming or the householders being absent. Eighteen households (0.7%) reported to have refused IRS, 15 of these gave someone being ill as the reason for refusal. Most households reported liking both IRS and ITNs (77%), 15% preferred ITNs and 7% preferred IRS.

Detailed information on net usage and ownership has been reported elsewhere [18]. The proportion of households with at least one ITN increased from 62.6% in the first survey to 90.8%. The mean number of ITNs owned per households increased from 1.2 to 2.1. The percentage of households with at least 1 ITN per two residents increased from 12.7 to 35.2% between the surveys. ITNs were reported to be used the previous night by 43% and 58% of study children in first and second survey respectively.

## Discussion

The results show that in Muleba during the first annual malaria transmission season the main risk factors for *P. falciparum* infection in children less than 15 years old were: house not being sprayed; historically high transmission in the cluster; low community wealth; low community coverage of ITN ownership and; child being aged 5–9 years old. During the second malaria season (after the UCC) the main risk factors associated with *P. falciparum* infection in children were: historically high transmission in the cluster; low household wealth; low community wealth and; age of child (<1 year olds at the lowest risk).

The protective effect of household IRS in the first malaria season was within the range reported by other studies [3], [7], [39], [40]. This may be an underestimate of the total effect of IRS in this area as this analysis did not investigate community protection provided by IRS since IRS coverage was universally high in the study area. There was weak evidence to suggest that the protection of IRS was greater in low transmission areas, but the number of households without IRS was too small to provide a stable comparator. This will be investigated further in the main trial where clusters were randomly allocated to receive IRS. No evidence was found for an association with *P. falciparum* infection and spray coverage after adjusting for other factors; coverage was high in all clusters and thus unlikely to be a concern. In the second malaria season when malaria prevalence was higher, household IRS status was not associated with *P. falciparum* infection. Lambdacyhalothrin has a residual effect of 3 to 6 months [41] and this could explain the absence of an impact five months after application. The effectiveness of IRS at controlling malaria has been shown to decrease with time since last spraying [37], [40], [42], [43]. Furthermore pyrethroid resistance has been reported in the area and could reduce the effectiveness of the IRS.

No association was seen between *P. falciparum* infection and reported individual use of an ITN the previous night. This finding is inconsistent with the vast amount of evidence from randomised control trials that showed the use of ITNs greatly reduced malaria morbidity and mortality [2]. Several reasons could explain this difference. Firstly, mosquito resistance to permethrin has been reported [45] and could have reduced the ITN effectiveness. Secondly, net use may have been miss-classified as found in Benin [46] where it was observed that net use reported by the household member was not associated with *P. falciparum* infection, while verified usage was associated with a lower odds of *P. falciparum* infection. Thirdly, there was no additional benefit to using nets when high coverage of IRS has been achieved as reported in Burundi [47]. Finally, these are observational data with the possibility of reverse causation; ITNs are possibly more likely to be used where the risk for malaria is greater [48]. Although adjustment for cluster sero-prevalence was included to allow for this, residual confounding may exist at the individual level.

While reported individual net use was not associated with a decrease in *P. falciparum* infection, clusters with higher community ITN coverage had lower malaria prevalence in the first survey. This suggests that ownership may be a better indicator for the protection given by ITNs than individual net use if self-reported ITN use is unreliable [49]. However, the association was not seen in the second survey after the UCC when ownership was generally higher. Ownership may have been more closely correlated with true usage in the first survey as the percentage of ITNs used was higher (81%) than in the second survey (64%).

During this analysis the combined use of IRS and ITNs was not compared to the use of one alone. There were few individuals that used a net alone and most net use (95%) was combined with IRS due to the high IRS coverage. Therefore we investigated the effect of each intervention adjusted for the other in multi-variable regression.

The apparent absence of personal protection provided by ITNs in this area with high IRS coverage suggests the need for trials comparing the combined use of IRS and ITNs compared to one alone. Current evidence as to whether there is an additional benefit from using ITNs in an area with high IRS is inconclusive [11], [12], [40], [47], [50], [51], [52], [53], [54]. The CRT for which this paper presents the baseline results, is intended to provide further evidence on this question.

In both surveys there was clear evidence of individual infection prevalence being associated with low socio-economic characteristics of the community. In survey two, in which household SES was available, it was shown that living in a poor household and a poor cluster were both independently associated with having increased odds of *P. falciparum* infection, highlighting the importance of both household and community level poverty. Although malaria is considered a disease of poverty [9], [55] a review of the effect of SES on malaria incidence reports that the evidence is mixed [56]. It has been hypothesised that there are three pathways for low household wealth to be associated with malaria: 1) less access to preventative measures; 2) less access to health care and; 3) increased risk of infection by: poor housing (mosquito entry), houses may be located nearer to mosquito breeding sites and higher susceptibility to illness especially due to poorer health and diet [57]. Access to healthcare was considered in this study but there was no evidence for an association between *P. falciparum* infection and the distance from the household to the nearest health facility after adjusting for other factors. It may be that it is not the actual distance but the accessibility due to poverty that is important, especially as health care and ACTs are not provided free of charge in Tanzania [1]. Household SES was generated from 11 components, three of which were household construction variables. When looking at these variables individually without adjusting for SES, grass roofing was associated with *P. falciparum* infection after adjusting for age and background malaria transmission; wall and floor material were not associated. Temu and collegues also found an association between grass roofing and malaria infection [58]. However, in this study it cannot be determined whether grass roofing was a risk factor in itself by providing shelter for mosquitoes, or whether it was just a good proxy for SES.

Infection prevalence was higher after the long rainy season (survey two prevalence = 22.8%) than after the short rainy season (survey one = 9.3%). However the estimates do not show the complete seasonal changes and may not capture the peak in cases. The 8.5% prevalence reported in children under five in Kagera region in the 2012 malaria indicator survey [59] is in line with the first survey results but lower than in the second survey. The prevalence estimates in this study and in the malaria indicator survey 2012 are lower than the 41% prevalence reported in children under five for the region from the malaria indicator survey between October 2007 and February 2008 [16]. Prevalence was heterogeneous between villages ranging from 1.3 to 61.5% in survey two. Heterogeneity between and within villages has previously been reported [60] and is particularly a feature of areas that have undergone control activities [61]. A similar range was reported on Bioko Island, Equatorial Guinea (5–72%) after 2 years of malaria control interventions [40]. The transmission heterogeneity in this area suggests it may be beneficial to target hotspots for more frequent or concerted malaria control [62].

The percentage of fever that can be attributed to *P. falciparum* infection was about 75% in both surveys. This suggests that malaria is responsible for a large proportion of the fever cases and thus disease burden in the area. The prevalence of symptomatic cases (fever and *P. falciparum* infection) was very similar between the two surveys as a higher percentage of the infected individuals had fever in the first malaria season. This could be explained if the second survey was conducted after the peak in symptomatic cases, but cannot be verified as the surveys were cross-sectional.

The level of any anaemia (55%) in our study was much lower than the estimates reported in the 2004–5 Demographic Health Survey in Kagera region (71%) [63]. This could be due to the reduction of malaria since the introduction of IRS.

IRS acceptance appears to be high even after six years of spraying as illustrated by very high reported coverage (95%) and that 95% of all households reported that they would accept IRS in the future. Most people appear to understand the purpose of IRS and this aids compliance. ITN ownership and usage has increased between the two surveys as reported elsewhere [18]. However, only 64% of ITNs were reported to be used the previous night in the second survey.

Sero-prevalence was used in this study to adjust for background malaria exposure and thus transmission levels when assessing the impact of the interventions. This is important in observational studies since uptake of the interventions may increase with risk of disease and the impact of IRS may differ between places of differing transmission intensities. The substantial heterogeneity in transmission in this study underscores the need for this adjustment. Sero-prevalence data has been used previously to adjust for malaria transmission intensity when evaluating the effectiveness of vector control interventions on Bioko Island [34], [37]. Given that sero-prevalence is an averaged exposure measure it is less susceptible to short term fluctuations in transmission and provides a valuable additional tool in monitoring and evaluating malaria control.

In conclusion, in this area where IRS coverage was high and LLIN distribution was equitable [18], poverty at both the community and household level were risk factors for malaria. This implies that it would be beneficial to target additional malaria control activities to poor households and communities, for example improving access to diagnosis and treatment. IRS was shown to be protective against malaria. However this was not sustained as there was no household level protection from IRS five months after application suggesting that this interval was greater than the residual life of the insecticide. It is possible that the low residual after five months was insufficient to kill pyrethroid resistant mosquitoes [44]. Higher community LLIN ownership was protective against malaria before the UCC when ownership was generally low. Reported LLIN usage was not associated with personal protection from malaria infection. The limited protection given by IRS and LLIN in some clusters suggests that it may be necessary to enhance malaria control interventions. This would include focusing on high transmission areas, a reduction of the interval between spray rounds, and switching to a non-pyrethroid insecticide for IRS. PMI changed its policy to spraying a carbamate in 2012 due to concerns about the effect of pyrethroid resistance on the effectiveness of IRS in the area.
